# Assessing the Quality and Reliability of Cardiac Rehabilitation Information on YouTube: A Systematic Evaluation

**DOI:** 10.7759/cureus.62752

**Published:** 2024-06-20

**Authors:** Huseyin Tezcan, Ezgi Akyildiz Tezcan

**Affiliations:** 1 Cardiology, Konya City Hospital, Konya, TUR; 2 Physical Medicine and Rehabilitation, Konya Numune Hospital, Konya, TUR

**Keywords:** quality of health care, video recording, social media, health information literacy, cardiac rehabilitation

## Abstract

Objectives: This study aims to systematically evaluate the quality and reliability of YouTube videos on cardiac rehabilitation, addressing a gap in the literature regarding the assessment of online health resources in this field.

Design and setting: The study is a cross-sectional analysis. This research was conducted entirely online, utilizing the YouTube platform for data collection.

Main measures: The videos were assessed for educational quality and reliability using modified versions of the DISCERN, Journal of the American Medical Association (JAMA), and Global Quality Scale (GQS) benchmarks. Specific data points such as upload date, length, uploader and narrator identity, and engagement metrics (views, likes, and dislikes) were also collected.

Results: Out of 300 videos initially reviewed, 140 met the inclusion criteria. The majority of videos were of low quality (67.9%), with medium (12.9%) and high-quality (19.3%) content being less common. Videos were predominantly uploaded by academic, university, or hospital sources (63.6%) and narrated by non-physician health professionals (41.4%). The content mainly provided general information about cardiac rehabilitation.

Conclusions: The study revealed a concerning predominance of low-quality YouTube content on cardiac rehabilitation, underscoring the necessity for healthcare professionals and academic institutions to enhance the quality of online resources.

## Introduction

Cardiovascular diseases are among the leading causes of death and disability worldwide [1]. Recent advancements in diagnostic techniques, enhanced management of risk factors, and improved acute care therapies have contributed to prolonged survival in patients with heart conditions [2,3]. However, this increase in longevity often means that these heart conditions are managed as chronic illnesses. This paradigm shift highlights the critical importance of cardiac rehabilitation in the ongoing management of these diseases.

Cardiac rehabilitation is an essential secondary prevention strategy, proven effective in reducing mortality and morbidity in individuals with cardiac ailments [4]. The World Health Organization (WHO) describes cardiac rehabilitation as a series of activities targeting the fundamental causes of cardiovascular disease [5,6]. It aims to improve patients' physical, mental, and social well-being, thereby empowering them to maintain or regain a vital role in society [5,6]. This multifaceted approach to cardiac rehabilitation includes five key components: nutritional counseling, risk factor modification, psychosocial management, patient education, and exercise training [4,7,8]. Originally introduced in the late 1960s, cardiac rehabilitation was initially recommended solely for low-risk patients who had survived an acute myocardial infarction [9]. However, over the past three decades, a growing body of evidence has underscored the benefits of cardiac rehabilitation. As a result, contemporary clinical guidelines now endorse the referral of a broader spectrum of cardiac patients to comprehensive cardiac rehabilitation programs. These include individuals recovering from acute myocardial infarction, percutaneous coronary intervention, coronary artery bypass surgery, heart valve repair or replacement, and heart transplant, as well as those living with chronic stable angina or systolic heart failure [10].

The digital era has seen a marked increase in the use of online platforms for patient education and health information dissemination [11]. Among the various platforms, YouTube stands out as a premier video hosting site, recognized for its role in health information dissemination and medical education [11,12]. It offers free access, enabling users to view, upload, and interact with videos on a myriad of topics [13]. YouTube's combination of audio and visual communication makes it exceptionally accessible to a wide demographic [14]. Although it serves as a powerful educational tool for health professionals to disseminate information and influence public health behaviors, the open-access nature of YouTube also presents challenges. The platform's allowance for any user to upload content introduces the risk of misinformation, a concern that is particularly significant in critical health areas like cardiac rehabilitation. This dual aspect of YouTube, as both an informative and potentially misleading platform, necessitates a cautious approach to its use for healthcare communication.

Despite the popularity of online health resources, the literature lacks studies evaluating the quality and reliability of YouTube content on cardiac rehabilitation. This study pioneers in filling this critical gap by providing an evidence-based evaluation of online resources, a first in the field of cardiac rehabilitation. We aim to systematically evaluate the quality and reliability of YouTube videos on cardiac rehabilitation, thereby offering crucial insights for patients seeking information online.

## Materials and methods

Study design and ethics

This study did not necessitate ethical clearance since it involved the analysis of publicly available online videos. Furthermore, the research excluded the involvement of any human subjects or animals.

Data collection

A systematic search was conducted on the YouTube platform (https://www.youtube.com/) on April 25, 2024. The search utilized the English terms "cardiac rehabilitation," "heart rehabilitation," and "cardiology rehabilitation." Prior to entering these keywords, the search history was cleared to ensure unbiased results. Videos that were not pertinent to cardiac rehabilitation - those that were duplicated, had unclear audio, or were in languages other than English - were excluded from the analysis. Additionally, videos lacking audio were also disregarded. The sorting of search results was done using "Relevance-Based Ranking." The initial 300 videos that met the criteria were then chosen for further examination, following the methodologies outlined in prior studies [12,15]. Each video selected in the preliminary round was downloaded, cataloged, and sequentially numbered for subsequent analysis. Post-selection, these videos underwent an eligibility check and were then presented to two expert reviewers for evaluation. The reviewers, specialists in cardiology and physical medicine and rehabilitation (PM&R), conducted independent assessments of the video content. In instances where the two reviewers' assessments differed, they jointly reviewed the video again to reach a consensus on the final score. Furthermore, to ascertain the consistency of the research, a kappa statistical test was conducted.

Video parameters

For each video, a set of specific data points was gathered: the upload date, the length of the video in seconds, the identity of the uploader, the identity of the narrator, and engagement metrics such as total views, likes, and dislikes. Additionally, the interaction rate was calculated using the formula: ((number of likes - number of dislikes) / total number of views) × 100%. The profiles of the uploaders were also documented and categorized into four distinct groups: academic, university, or hospital; society/non-profit organization; news organizations, and independent users. Also, the profiles of the narrators were also documented and categorized into four distinct groups: physician, non-physician health professional, patient, and others.

Educational quality and reliability of the videos

For the purposes of this research, modified versions of the DISCERN [16], the Journal of the American Medical Association (JAMA) [17], and the Global Quality Scale (GQS) [18] benchmarks were employed. The videos were evaluated individually, and an average score for each was determined.

This study utilized a modified version of the original DISCERN questionnaire to evaluate the quality and reliability of health information presented in the videos. This modified tool comprises five questions, with the response options limited to "yes" or "no." Each affirmative response is scored as one point, making the highest possible score five. The questions are as follows: (1) Is the video clear and achieved? (2) Are reliable sources of information used? (3) Is the information presented balanced and unbiased? (4) Are additional sources of information listed for reference? (5) Are uncertain areas mentioned? In this modified DISCERN system, a reliability score of 3 or above is considered indicative of high reliability.

The JAMA evaluation method is applied to analyze online videos and resources, focusing on four key aspects: authorship, attribution, disclosure, and currency, with each aspect valued at one point. For authorship (1 point), it's essential that the video includes details of its authors, contributors, and contact information. Regarding attribution (1 point), proper citation of references and sources is required. Disclosure (1 point) involves openly stating any conflicts of interest, funding sources, sponsorships, advertisements, support, and ownership of the video. Last, currency (1 point) requires that the video clearly state its publication and, if applicable, update dates. A cumulative score of 4 in this assessment signifies that the resource is of high quality.

Additionally, the GQS was employed to assess each video. This involved using a 5-point Likert scale to judge the overall quality and utility of the information presented in the videos. The GQS ratings are as follows: 1 signifies poor quality with minimal information and low utility for patients; 2 indicates generally poor quality with some useful content; 3 represents moderate quality where key information is adequately presented; 4 denotes good quality with comprehensive and patient-helpful content; 5 reflects excellent quality with outstanding informational value for patients. A score of 5 represents the pinnacle of quality, while a score of 1 indicates the lowest. Based on these scores, the videos are categorized into three quality levels: low quality (1-2 points), medium quality (3 points), and high quality (4-5 points).

Statistical analysis

We utilized IBM SPSS Statistics for Windows, Version 22 (Released 2013; IBM Corp., Armonk, New York, United States) for our data analysis. To determine data normality, we applied the Shapiro-Wilk test, which revealed a non-normal distribution of the data. Demographic information was analyzed using standard descriptive statistics, including frequency, percentage, mean, and standard deviation or median with interquartile ranges. Due to the non-normal distribution in all groups, the Kruskal-Wallis test was employed to compare the medians across the groups. Significant differences identified by the Kruskal-Wallis test led to post-hoc pairwise comparisons using the Mann-Whitney U test with Bonferroni correction. For categorical data comparisons, the chi-square test was the method of choice. The Kappa coefficient was used to assess inter-rater and intra-rater agreement. Our findings were considered significant at a 95% confidence interval and a significance threshold of p < 0.05. For the Bonferroni correction, we deemed a p-value below 0.017 (0.05/3) as statistically significant.

## Results

In this study, we initially reviewed 300 videos. Following a thorough examination and the application of our exclusion criteria, which included the removal of 2 non-English videos and 2 videos not relevant to cardiac rehabilitation, a total of 140 videos were deemed suitable for evaluation. The general characteristics of these videos, along with their quality and reliability scores, are detailed in Table 1.

**Table 1:** Video features, quality, and reliability scores of videos JAMA: Journal of the American Medical Association

Parameter	Median (IQR)
Duration (seconds)	259 (152, 693)
Time since upload (days)	1398 (837, 2454)
Number of views	3270.5 (916, 15780.75)
Number of likes	14.5 (3, 78.5)
Number of comments	0 (0, 2)
Interaction rate	0.007 (0.003, 0.110)
Modified DISCERN	3 (2, 3)
JAMA	1 (1, 2)
Global Quality Scale	2 (1, 3)

Upon assessing the videos using the GQS, it was found that 67.9% of the videos were classified as low quality, 12.9% as medium quality, and 19.3% as high quality. Kappa coefficient calculation showed a value of 0.82 (95% CI (0.78, 0.86)) for inter-rater reliability. For the first author, the intra-rater reliability was found to be 0.87 (95% CI (0.83, 0.91)) and for the second author, the intra-rater reliability was 0.89 (95% CI (0.85, 0.93)).

The majority of the video content originated from academic, university, or hospital sources (63.6%), and the narrators were predominantly non-physician health professionals (41.4%). The videos primarily provided general information about cardiac rehabilitation. Detailed information about the sources of the videos, the profiles of the narrators, and the specific content of the videos can be found in Table 2 and Table 3, respectively. The video source (p=0.103), narrator (p=0.259), or quality degree (p=0.268) was not found to be associated with the number of views. A comparison of video features according to quality classification can be found in Table 4.

**Table 2:** Video sources and narrators

Video sources	Number (percentage)
Academic/university/hospital	89 (63.6%)
Society/non-profit organization	33 (23.6%)
News agency	7 (5%)
Independent user	11 (7.9%)
Video narrators	
Physician	53 (37.9%)
Non-physician health professional	58 (41.4%)
Patient	9 (6.4%)
Other	20 (14.3%)

**Table 3:** Video contents

Video contents	Number (percentage)
General information about cardiac rehabilitation	103 (73.6%)
Exercise	20 (14.3%)
Patient experience	10 (7.1%)
Other	7 (5%)

**Table 4:** Comparison of video features according to quality classification ^*^Post-hoc pairwise comparisons significance occurred between low and medium quality, low and high quality, medium and high quality. ^†^Post-hoc pairwise comparisons significance occurred between low and high-quality. ^‡^Post-hoc pairwise comparisons significance occurred between low and medium quality. ^§^Post-hoc pairwise comparisons significance occurred between low and medium quality, low and high quality JAMA: Journal of the American Medical Association

	Low quality	Medium quality	High quality	p-value
Video sources				0.258
Academic/university/hospital	58 (65.17%)	10 (11.24%)	21 (23.6%)	
Society/non-profit organization	26 (78.79%)	3 (9.09%)	4 (12.12%)	
News agency	5 (71.43%)	2 (28.57%)	0	
Independent user	6 (54.55%)	3 (27.27%)	2 (18.18%)	
Video narrators				0.051
Physician	34 (64.15%)	6 (11.32%)	13 (24.53%)	
Non-physician health professional	39 (67.24%)	6 (10.34%)	13 (22.41%)	
Patient	9 (100%)	0 (0%)	0 (0%)	
Other	13 (65%)	6 (30%)	1 (5%)	
Modified DISCERN	2 (1, 3)	3 (3, 3)	4 (4, 4.5)	p<0.01^*^
JAMA	1 (1, 2)	1 (1, 2)	2 (2, 3)	p<0.01^*^
Duration of video	200 (119, 435)	294 (180, 903.25)	555 (291.5, 1049)	p<0.01^†^
Number of views	2941 (792, 14410.5)	6856 (1997, 22130.25)	4261 (1499, 18401)	p=0.268
Number of comments	0 (0, 1)	1 (0, 4.75)	0 (0, 4)	p=0.0128^‡^
Number of likes	11 (3, 43.5)	48 (16.5, 193.75)	37 (11.5, 153.5)	p<0.01^§^
Interaction rate	0.01 (0, 0.01)	0.01 (0.01, 0.01)	0.01 (0.01, 0.01)	p<0.01^§^
Views per day	1.87 (0.64, 6.86)	4.75 (1.83, 21.36)	2.71 (1.46, 16.36)	p=0.0597
Comments per day	0 (0, 0)	0 (0, 0.01)	0 (0, 0.01)	p=0.0665
Like per day	0.01 (0, 0.02)	0.045 (0.02, 0.325)	0.03 (0.01, 0.12)	p<0.01^§^

## Discussion

This study represents a pioneering effort to systematically evaluate the quality and reliability of YouTube videos on cardiac rehabilitation. Our findings present a significant presence of low-quality content (67.9%) in the realm of cardiac rehabilitation on YouTube. This highlights a concerning aspect of online health information.

In a prior assessment of YouTube content relating to cardiology, it was observed that a majority of videos concerning myocardial infarction were of high quality [19]. In addition, 63% of the videos pertaining to hypertension were deemed useful [20], and 53% addressing aortic valve stenosis were classified as such [21]. Given that videos rated as GQS 2 can also be considered beneficial, for including minimal beneficial information, the findings from the studies on hypertension and aortic valve stenosis align with the results of our current research. Nonetheless, the notable disparity in the quality of videos on myocardial infarction merits further investigation. We hypothesize that the variance may be attributed to differences in general awareness between the topics. Cardiac rehabilitation, unlike myocardial infarction, might be a subject that is not adequately comprehended by healthcare professionals and patients alike. This hypothesis is supported by the fact that only one in four patients who require cardiac rehabilitation services actually receive them [22]. Such a disparity in understanding and accessibility underscores the need for more targeted educational efforts in this area.

Upon examination of the online video content pertaining to various rehabilitation domains, a recurring theme emerges. Studies dedicated to the evaluation of online resources in vestibular rehabilitation [23], cancer rehabilitation [12], stroke rehabilitation [24], and pulmonary rehabilitation in the context of chronic obstructive pulmonary disease [25] consistently report that these videos are predominantly of low quality and are characterized by a paucity of comprehensive information. This trend underscores a pervasive issue of substandard quality within rehabilitation-related video content available online. Such findings highlight a critical gap in the provision of high-quality, informative digital resources in these specialized areas of rehabilitation, indicating a pressing need for improvement to support patient education and public health awareness.

Moreover, our investigation also revealed the presence of high-quality videos, albeit in a lesser proportion. This finding suggests a significant opportunity for healthcare professionals and academic institutions to contribute more rigorously vetted and superior-quality content to platforms such as YouTube, thereby mitigating the widespread issue of misinformation. In terms of viewer engagement, as indicated by likes and interaction rates, videos of medium to high quality demonstrated superior performance compared to those of lower quality. This trend offers a promising outlook for the impact and reach of videos of medium and high quality.

A recent study using Google Trends analysis demonstrated a notable surge in internet searches related to cardiovascular diseases during the pandemic period. This trend underscores the critical need for reliable and accurate online health information, as more individuals turn to digital platforms for guidance on managing their health conditions. Our study's findings on the variable quality of YouTube videos on cardiac rehabilitation highlight this pressing issue. Ensuring the availability of high-quality, trustworthy online resources is essential to support patients in making informed decisions about their health, particularly in the post-COVID-19 pandemic era [26].

The study presents several limitations that should be considered. First, all the videos were assessed cross-sectionally within a single timeframe. Given that these evaluations were not conducted simultaneously, it's possible that the number of views, likes, dislikes, and comments on the videos may have fluctuated. Moreover, new videos might have been added, and some videos could have been removed, potentially impacting the results. Another limitation, while the inter-rater compliance scores were high, it's worth noting that the assessment by just two physicians might not provide a comprehensive evaluation. A larger and more diverse panel of raters could have enhanced the study's validity. This study's scope was limited to English-language videos, potentially excluding high-quality content in other languages. Future research could extend to multilingual content to provide a more global perspective on cardiac rehabilitation resources available on YouTube. Finally, it's essential to recognize that video quality assessments inherently contain a subjective element. This subjectivity may introduce a degree of variability in the findings. On a positive note, a strength of our study lies in its interdisciplinary approach. Both a cardiologist and a PM&R specialist participated in the evaluation of the videos, providing diverse perspectives and expertise.

## Conclusions

In conclusion, this study, a first in its field, reveals a concerning predominance of low-quality YouTube content on cardiac rehabilitation, with 67.9% of videos evaluated falling into this category. Despite this, the presence of some medium- and high-quality videos highlights an opportunity for healthcare professionals and academic institutions to improve online resources. Our findings emphasize the need for more reliable and informative content in this digital era, where online platforms are increasingly becoming key sources of health information. The collaborative involvement of a cardiologist and PM&R specialist in this study underlines the importance of interdisciplinary approaches in enhancing patient education and public health awareness. Ultimately, this research underscores the critical need for the medical community to actively engage in producing and disseminating high-quality, accurate content on cardiac rehabilitation, ensuring that patients and the public have access to trustworthy and helpful resources.
